# Nursing in the Age of Artificial Intelligence: Protocol for a Scoping Review

**DOI:** 10.2196/17490

**Published:** 2020-04-16

**Authors:** Christine Buchanan, M Lyndsay Howitt, Rita Wilson, Richard G Booth, Tracie Risling, Megan Bamford

**Affiliations:** 1 Registered Nurses' Association of Ontario Toronto, ON Canada; 2 Arthur Labatt Family School of Nursing Western University London, ON Canada; 3 College of Nursing University of Saskatchewan Saskatoon, SK Canada

**Keywords:** nursing, artificial intelligence, machine learning, robotics, compassionate care, scoping review

## Abstract

**Background:**

It is predicted that digital health technologies that incorporate artificial intelligence will transform health care delivery in the next decade. Little research has explored how emerging trends in artificial intelligence–driven digital health technologies may influence the relationship between nurses and patients.

**Objective:**

The purpose of this scoping review is to summarize the findings from 4 research questions regarding emerging trends in artificial intelligence–driven digital health technologies and their influence on nursing practice across the 5 domains outlined by the Canadian Nurses Association framework: administration, clinical care, education, policy, and research. Specifically, this scoping review will examine how emerging trends will transform the roles and functions of nurses over the next 10 years and beyond.

**Methods:**

Using an established scoping review methodology, MEDLINE, Cumulative Index to Nursing and Allied Health Literature, Embase, PsycINFO, Cochrane Database of Systematic Reviews, Cochrane Central, Education Resources Information Centre, Scopus, Web of Science, and Proquest databases were searched. In addition to the electronic database searches, a targeted website search will be performed to access relevant grey literature. Abstracts and full-text studies will be independently screened by 2 reviewers using prespecified inclusion and exclusion criteria. Included literature will focus on nursing and digital health technologies that incorporate artificial intelligence. Data will be charted using a structured form and narratively summarized.

**Results:**

Electronic database searches have retrieved 10,318 results. The scoping review and subsequent briefing paper will be completed by the fall of 2020.

**Conclusions:**

A symposium will be held to share insights gained from this scoping review with key thought leaders and a cross section of stakeholders from administration, clinical care, education, policy, and research as well as patient advocates. The symposium will provide a forum to explore opportunities for action to advance the future of nursing in a technological world and, more specifically, nurses’ delivery of compassionate care in the age of artificial intelligence. Results from the symposium will be summarized in the form of a briefing paper and widely disseminated to relevant stakeholders.

**International Registered Report Identifier (IRRID):**

DERR1-10.2196/17490

## Introduction

### Artificial Intelligence

Artificial intelligence (AI) has been defined as “the theory and development of computer systems [which are] able to perform tasks that normally require human intelligence, such as visual perception, speech recognition, decision-making, and translation between languages” [1]. Machine learning and deep learning are subsets of AI and have become popular neologisms used to describe various, more specific algorithmic methodologies and techniques used to process information in ways that can imitate human-like decision making [2]. Within health care, the use of technology possessing AI has become increasingly popularized due to its capacity to sort through, analyze, and find patterns among large amounts of research evidence and patient data, ultimately discovering new meaning [3]. Current examples of where AI has been integrated within health care include clinical decision support systems [4,5], virtual nurses [6], and social robots with natural language processing abilities [7]. Furthermore, researchers are currently exploring the use of deep learning for diagnostic purposes and prediction of future clinical events [8], and machine learning is being used to understand past patient experiences, while constantly changing and updating as new data become available [3]. Given the enormity of the financial investments in these technologies made to date, it is predicted that health care services and delivery will be transformed in significant ways in the coming decade; authors have suggested that global spending on digital health technologies that incorporate AI will exceed $36 billion by 2025 [9]. In addition, the paradigm shift in preparing nurses for the digital future is already being discussed; one recent publication suggests that AI-driven technologies need to be considered as a new means of addressing health care challenges in the 21st century and that the health care workforce needs to be prepared for these changes accordingly [10]. Although AI is still a nascent topic, emerging literature has suggested that digital health technologies that use elements of AI will begin to impact the daily aspects of people’s lives in the not-too-distant future [11,12].

### Background

AI-driven digital health technologies that have decision-making capacities independent of humans are currently being used in numerous health care organizations. For example, early warning systems and clinical decision support systems that utilize machine learning principles are being used to aid nursing workflow and provide more personalized patient care in hospital settings [11]. Virtual nurses are another example of a digital health technology that incorporates AI. Virtual nursing avatars are accessible through a patient’s computer or smartphone device and enable health care organizations to collect patient information, provide discharge instructions, coach patients, and assess patient health status from a remote location [6]. In addition, there is a growing body of literature exploring the use of intelligent assistive technological devices to support older adults that live alone with various activities of daily living [13]. Such devices have the capacity to sense and respond to consumer needs, and their intelligent nature allows them to operate autonomously within a network of related devices [13]. Not only are these technologies relevant to older adults in the home setting but they can also be translated for use in other health care contexts, such as long-term care residences and rehabilitation centers [7]. Finally, in Canada, Humber River Hospital has incorporated a fully digital infrastructure that includes the use of AI-driven robots to support children and their families as part of the Child Life Program [14]. These examples demonstrate the capabilities of AI and the ways in which this technology is already starting to transform health care systems.

While AI is frequently used in health care to assist with data analytics and clinical decision making [11], the potential for AI-driven digital health technologies to influence the relationship between nurses and their patients must not be understated. As examined by Idhe’s philosophy of technology [15], there is invariably a relationship between human beings, technology, and the surrounding world; whether technology is working in the background of our daily lives or through embodied relationships, there are numerous human-technology relationships that can be explored further [15]. Thus, this scoping review is intended to further one’s understanding of how AI-driven health technologies influence the nurse-patient relationship and nurse work in general.

Within the nursing profession, the delivery of compassionate care is a core and valued historical tenet of nursing theory and practice [16-18]; providing safe, compassionate, competent, and ethical care is a core value of nursing practice, as reflected in numerous international nursing practice frameworks [19-21]. Compassionate care has been described as an empathetic response to suffering that involves person-centered care, meaning treating individuals the way they want to be treated [22]. It can be expressed by nurses using silent presence, active listening, firm touch, a caring and respectful attitude, and a kind manner [23]. Compassionate care involves the relationships among health care providers and their patients and can be influenced by a health systems’ infrastructure [3]. With compassionate care as a core tenet of the nursing profession, it is important to reflect on the future influence of AI-driven digital technologies on nursing care.

### Goals of the Review

**Figure 1:**
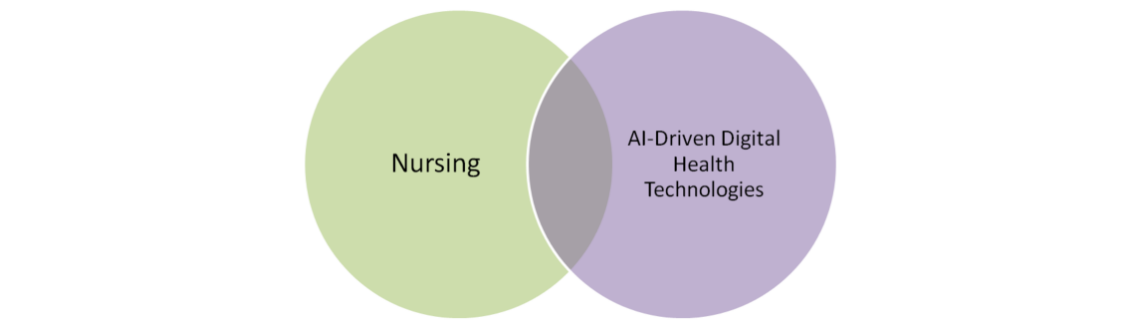
Main concepts explored in the review.

It is anticipated that emerging trends in AI-driven digital health technologies will change the nature of nursing roles and practice [2], and in light of technological advancements, nurses will need to ensure the continued delivery of compassionate nursing care [3]. As such, it is important to understand how AI-driven digital health technologies are changing nursing roles and affecting patient and caregiver experiences with nursing care. Nursing students and licensed nurses will need to be equipped to provide care in a technological world, and the influence of AI-driven digital health technologies on nursing education will require examining. Finally, to ensure that AI-driven digital health technologies promote compassionate care (as a core tenet of nursing care) rather than hinder it, it will be important to understand how nurses are involved in the co-design of AI-driven digital health technologies. To explore these focus areas, this scoping review aims to summarize the findings of 4 research questions that explore the relationships between nurses, patients, and AI-driven digital health technologies (see Figure 1). Furthermore, the findings from this scoping review will be used to reflect on how emerging trends may impact nurses’ delivery of compassionate care. The results of this scoping review and the subsequent reflections will be disseminated in a briefing paper and will inform a symposium on the topic of nursing and compassionate care in the age of AI. For this review, a scoping methodology is appropriate due to its exploratory nature and the current literature gap on this research topic. As stated by Tricco et al [24], scoping reviews aim to synthesize evidence and assess the scope of literature on a topic, which is the objective of this review due to the emerging nature of this topic. 

## Methods

### Scoping Review

Standardized reporting guidelines outline items that should be included in research reports to enhance methodological transparency [24]. This protocol was developed using guidance for relevant items from the Preferred Reporting Items for Systematic Reviews and Meta-Analyses Extension for Scoping Reviews (PRISMA-ScR) [24], as a protocol guidance document for scoping reviews has not been developed. This scoping review is registered in the Open Science Framework database (registration doi: 10.17605/OSF.IO/RTFJN) [25].

This scoping review follows the methodological framework proposed by Arksey and O’Malley [26] and further advanced by Levac et al [27]. This framework delineates 6 steps to map the extent and range of material on a research topic [27], providing clarity on what is known and not known on a topic and situating this within policy and practice contexts [28].

### Step 1: Identify the Research Question

A steering committee of experts in digital health technologies was assembled to inform the research questions that would best meet the overarching objective of this review. The steering committee consists of advanced practice nurses, nurse researchers, educators, a patient advocate, registered practical nurses, and registered nurses from various health care sectors. This steering committee is co-chaired by 2 doctorally-prepared nurses who possess independent programs of research in digital health and nursing (RB and TR). Additionally, the Associate Director of the Registered Nurses’ Association of Ontario (RNAO) Guideline Development and Evaluation team (MB) and RNAO eHealth Program Manager (RW) were consulted during the development of the research questions.

Levac et al [27] recommend scoping review questions be broad in nature; however, concepts should be clearly articulated to establish an effective search strategy. Using these recommendations, the 4 research questions explored in this review were derived through steering committee discussion and consensus. Due to timeframes and feasibility, the number of research questions was limited to 4:

1. What influences do AI-driven digital health technologies have, or are predicted to have, on the patient/caregiver experience of compassionate care delivered by nurses?
2. What influences do emerging trends in AI-driven digital health technologies have, or are predicted to have, on all domains of nursing practice (ie, administration, clinical care, education, policy, and research)?
3. What influences do emerging trends in AI-driven digital health technologies have, or are predicted to have, on nursing education across all domains?
4. What involvement do nurses have, or are predicted to have, in the co-design of AI-driven digital health technologies?

For the purposes of this scoping review, digital health technologies refer only to those technologies that meaningfully incorporate AI. Literature focused on electronic medical records, telehealth systems, genomics, virtual reality devices, and other technologies that do not actively utilize a discernible or definable form of AI will be excluded. For a technology to be identified as “emerging,” the author will have used the word “emerging” or a synonym (eg, new, innovative) to describe the technology, or the co-chairs or steering committee will have identified the technology as emerging based on experience in the field. In this review, a nurse refers to any nurse (eg, registered nurse, registered practical nurse, licensed practical nurse, nurse practitioner) working in any of the 5 nursing domains outlined by the Canadian Nurses Association: administration, education, clinical practice, policy, and research [19]. These 5 nursing domains are also applicable to international nursing settings, as identified in similar international nursing practice frameworks [20,21].

### Step 2: Identify Relevant Studies

#### Peer-Reviewed Literature

The databases MEDLINE, Cumulative Index of Nursing and Allied Health Literature (CINAHL), Embase, PsycINFO, Cochrane Database of Systematic Reviews, Cochrane Central, ERIC, Scopus, Web of Science, and Proquest were searched for peer-reviewed literature using searches developed by an information specialist. The search terms (Multimedia Appendix 1) and inclusion/exclusion criteria (Textbox 1) were developed by examining relevant publications and through consultation with the co-chairs, steering committee, and information specialist. Terms relating to “compassionate care” specifically were not included in the search strategy. Through discussion, it was decided that including “compassionate care” as a search term would narrow the yield of results; as stated by Levac et al [27], scoping reviews are broad in nature as the focus is on summarizing the breadth of the evidence. Therefore, the reviewers felt it was important that the search strategy was broad to encompass a large yield of studies. For the purposes of this review, electronic medical records were also excluded since they are a well-established technology and not an emerging trend. Clinical information from electronic medical records that utilize AI-driven technology (ie, advanced clinical decision support systems) will be included. Due to feasibility, timeframes, and the lack of detailed conference proceedings provided, it was decided that they would also be excluded.

**Inclusion and exclusion criteria.**

**Inclusion Criteria**

- Relates to one of the research questions
- Focuses on AI-driven digital health technology
- Clear connection to nursing (can broadly focus on health care workforce but must be transferable to nursing)
- Published after January 1, 2014 (unless it is a seminal article)
- Peer-reviewed or grey literature, including any research study design (ie, randomized controlled trials, systematic reviews and quasi-experimental, observational and qualitative designs), thesis/dissertations, discussion papers, or white papers
- Printed in English
- Accessible for retrieval

**Exclusion Criteria**

- Studies/articles not relevant to one of the research questions
- No clear focus on AI-driven digital health technologies
- Focus on electronic medical records
- Specific to other health care profession (eg, physicians only)
- Articles published before January 2014 (unless it is a seminal article)
- Conference proceedings
- Clinical trials
- Not available in English

A test search was run in MEDLINE, and the first 100 articles were reviewed by the reviewers and co-chairs for relevancy. Any additional search terms identified as per relevant articles were added to the search string at this stage. Once consensus was reached among the reviewers and co-chairs regarding relevance of the articles in the test search, it was decided that the full search would be run in the remainder of the databases.

Qualitative and quantitative studies were eligible for inclusion. Due to the rapid increase in use of digital health technologies over the last 10 years, initially it was decided that only literature published after January 1, 2009 would be included. However, due to the substantial yield of articles, it was not feasible to screen all articles considering the project timeframes, and the limit was reduced to the last 5 years upon consultation with the committee co-chairs. Additionally, to balance breadth and feasibility when conducting this scoping review, books/book chapters and conference proceedings were excluded, and only literature published in English was included. Keywords related to the 2 main concepts (“artificial intelligence” and “nursing”) were used to search databases (see Multimedia Appendix 1). As already stated, to allow for a broader search, “compassionate care” was not used as a search term in the database search; however, when reflecting on the findings, the reviewers utilized an analytic lens examining the literature’s relevance to compassionate care, as this is one of the main focuses of the scoping review. Reviewers referred to the definition of compassionate care outlined in the introduction of this paper when assessing each article’s relevance to compassionate care.

#### Grey Literature

Grey literature was retrieved by searching Proquest, CINAHL, and PsychINFO for English theses and dissertations as well as discussion papers written after January 1, 2014. The same search terms used to search the electronic databases for peer-reviewed literature were used. In addition, targeted website searches of the following websites will be conducted to retrieve relevant white papers: World Health Organization, National Health Service, Office of the National Coordinator for Health Information Technology, Institute for Research on Healthy Public Policy, Canada Health Infoway, Canadian Association of Schools of Nursing, and Healthcare Information and Management Systems Society. The targeted websites were discussed and agreed upon by the steering committee. Targeted website searches will be performed using Google search strings developed by the information specialist and run by the reviewers (CB and LH).

All peer-reviewed and grey literature results will be downloaded into EndNote X7.8 (Clarivate Analytics, Philadelphia, PA) and imported into the Web-based systematic review software Distiller SR (Evidence Partners, Ottawa, Canada) for review. The expert steering committee will also be asked to identify other potentially relevant peer-reviewed and grey literature materials not identified through prior search strategies (ie, “hand-searched” articles).

### Step 3: Study Selection

A screening guide developed by the reviewers (CB and LH) will be used to determine if the inclusion and exclusion criteria have been met (Textbox 1). Feedback was obtained from the co-chairs (RB and TR), eHealth Program Manager and project lead (RW), and Associate Director of the Guideline Development and Evaluation team (MB) while developing the screening guide. The 2 reviewers will independently pilot test the screening guide and review the first 100 abstracts before continuing with screening. Results will be discussed, and revisions to the screening guide will be made as needed. An example of an included article and an excluded article will also be presented to the project team to ensure appropriateness of the articles being included. All titles and abstracts will be independently screened by the 2 reviewers using the screening guide, and the reviewers will meet at the beginning, middle, and final stages of the screening process to discuss challenges related to study selection. First, titles and abstracts will be screened for relevance to “AI-driven digital health technologies” and “nursing” and the general inclusion criteria (Textbox 1). Next, any included full-text articles will be independently reviewed by the 2 reviewers for relevance to determine which of the 4 research questions they address. There will be specific inclusion questions in alignment with each of the research questions to direct the reviewer to which question the article could fall under. Discrepancies in study selection will be resolved by consensus, with final decisions being made by the project lead (RW) if consensus between the 2 reviewers cannot be reached. When screening the full-text studies, reviewers will keep in mind person-centered care principles [29], as these are an important element of nursing practice and compassionate nursing care.

### Step 4: Charting the Data

The data charting process will start with studies related to research question 1 and follow in a sequential order (research question 2, then 3, lastly question 4). Draft data charting forms will be developed in Microsoft Excel 2007 (Microsoft Corp, Redmond, WA) for each research question by the 2 reviewers. This form will be reviewed by MB, and the final form will be approved prior to pilot testing. The 2 reviewers (CB and LH) will pilot test the forms by independently charting the data from a representative sample (ie, 5-10 articles per research question) to ensure that consistency is achieved. This sample data charting form will be shared with the co-chairs (RB and TR), and further refinements will be made. Once consistency is achieved and the pilot-tested forms are approved, data from each included full-text article will be charted by one member of the research team and verified by a second member to ensure all relevant data are charted. Levac et al [27] suggest that the development of charting forms is an iterative process and the forms are expected to evolve as literature is reviewed and findings important to the research questions are added to the data fields.

### Step 5: Collating, Summarizing, and Reporting the Results

Levac et al [27] recommend that scoping reviews provide a numerical summary of the types of literature retrieved and a descriptive thematic summary of themes arising. Given the expected diverse body of literature, categorical data related to specific elements (ie, study methods, context of study, aim and purpose, key findings) will first be recorded (see Textbox 2); this information will align with the data charting forms for each research question. Study findings will then be synthesized using narrative description. Outcomes will be reported by study type for each research question (ie, qualitative versus quantitative study designs), and themes that emerge for each question will be reported. The findings will also be collated in relation to domains of nursing practice, in order to identify gaps for future research considerations. Pending the results, visual representations of the data may also be created. NVivo 12 (QSR International Pty Ltd, Burlington, MA) and Microsoft Excel 2007 (Microsoft Corp, Redmond, WA) will be used to assist with categorizing and analyzing the data as appropriate.

**Sample data charting elements.**

**Article Information**

- RefID number
- Data charted by (initials)
- Author
- Year
- Study design
- Country
- Aim/purpose

**Population**

- Nursing designation (registered nurse [RN], registered practical nurse [RPN], licensed practical nurse [LPN], nurse practitioner [NP], student)
- General description of “health care providers”
- Domain of practice or setting

**Intervention**

- Type of artificial intelligence–driven digital health technology discussed
- Brief description of study

**Study Findings**

- Key findings related to the research questions
- Relevance to compassionate care

### Step 6: Consultation

Levac et al [27] list consultation as a final and mandatory step in the scoping review process. Feedback will be sought from the co-chairs throughout the scoping review process. Once preliminary findings have been identified, a document summarizing the articles included and the themes identified for each research question will be circulated to the steering committee to review. During the consultation, the steering committee members will be asked to reflect on whether the themes identified resonate with their areas of expertise or if there were any themes they expected to see that were not identified. This consultation with the co-chairs and steering committee will occur through video conference call meetings and email. Upon completion of the review, a symposium with key partners and stakeholders will be held to further consult and discuss the findings, foster new delivery models of compassionate care involving digital health technologies, and help build leadership capacity among health care providers.

## Results

Electronic database searches were conducted in November 2019, and 14,415 results were retrieved. When the search was limited to the last 5 years, a total of 10,318 articles were retrieved. Title and abstract screening, data charting, and the remaining steps of the scoping review including dissemination (ie, symposium and subsequent briefing paper) aim to be completed by the fall of 2020. Preliminary screening results show that social robots that utilize AI are being used more frequently in long-term care settings with elderly patients [30,31], which may influence the therapeutic relationship between patients and their nurses. Furthermore, clinical predictive models using AI-driven technologies are becoming more advanced and have the potential to positively impact patient care [32,33]; nurses are increasingly becoming involved in the development of these AI-driven digital health technologies [2,12].

## Discussion

### Preliminary Findings

Digital health technology is frequently conceptualized as being at odds with humanistic, compassionate care, yet both play important roles in the delivery of health care [34]. This dualistic portrayal may prevent health care providers from recognizing the ways in which technology is ubiquitous in their own clinical practice, shaping their relationships with patients in ways that can be difficult to see [34]. The entwinement of technology in health care creates risks and possibilities; health care providers can use technology positively in the provision of patient care, and they can also become dependent on technology to the extent that they lose their humanness [34-36]. Without critical recognition of their entwined human and technology relationships, health care providers are at risk of continuously struggling against technology or being governed by it [34]. However, with conscious recognition of the ways in which technology is enmeshed in clinical practice, health care providers can preserve their humanness, recognize the embodied experiences of patients, and ensure compassionate care is actualized in a technological world [34].

Compassionate care is fundamental to the nursing profession [16-18], and without this core tenet, the nursing profession as it exists now may cease to remain. By critically examining the influence of technology on nursing practice and, more specifically, the influence of AI on compassionate nursing care, the profession can plan for its future trajectory in a technological world.

After collating and analyzing the findings of this scoping review, a briefing paper will be written. The briefing paper will summarize the findings in a narrative fashion, organized in a way that addresses the implications for nursing, health care policy, and future research. A manuscript containing the final analysis will be written and submitted for publication, and the PRISMA-ScR will be completed and submitted with the paper [24].

### Limitations

One limitation of this scoping review was the inability to search engineering and computer science databases, due to accessibility issues and organizational licensing restrictions; this limitation may lead to some gaps in the research findings. Future research encompassing engineering and computer science databases on this topic is advised.

In addition, this scoping review did not incorporate the Peer Review of Electronic Search Strategies elements [37]. This process involves peer review of the work of the information specialist, to ensure that the search strategy is appropriate [37]. Typically, this process is only done for systematic reviews, and due to the chosen methodological framework [27] along with timeframes and feasibility, it was not conducted for this review.

Furthermore, the reviewers used percentage agreement when calculating interrater agreement during title and abstract screening, for feasibility purposes, with a percentage agreement rating of 97%. However, it is recognized that this is not as reliable as Cohen’s kappa; in future projects, the reviewers will consider using Cohen’s kappa for greater interrater reliability.

The lack of quality assessment of the included articles is recognized as another limitation of this scoping review. However, scoping reviews typically do not appraise the quality of evidence in the primary research studies [26]. Furthermore, scoping reviews do not synthesize the research findings based on the relative quality of evidence in favor of a particular intervention; instead, they offer a narrative or descriptive account of the evidence [26]. Although these are potential limitations, scoping reviews can provide a large breadth of research studies in a relatively short amount of time, allowing reviewers to map out the gaps in research as well as summarize and disseminate their findings in a realistic and feasible manner [26]. Finally, given the emerging nature of this topic, performing a quality appraisal may exclude research studies that are more discussion-based or qualitative in nature, thus leading to very limited results.

### Conclusions

From a historical perspective, to consider the work of Sandelowski [38], nurses can take one of two viewpoints on technology: technological optimism or technological romanticism. Technological optimism encompasses a positive viewpoint, where one sees the benefits of technology on nursing practice; conversely, nursing romanticism holds a more negative viewpoint, where technology is seen as disruptive and dangerous to nursing practice [38]. In recent years, technology has become increasingly entrenched in nursing practice. Although Sandelowski’s viewpoints may not necessarily be true of today’s nurses, it is important to recognize both of these perspectives in order to ensure compassionate care is actualized as AI-driven digital health technologies continue to emerge.

Healthcare technology and compassionate care both play vital roles in the provision of nursing care. To our knowledge, this is the first scoping review to examine the influence of emerging trends in AI-driven digital health technologies on all domains of nursing and, more specifically, on the compassionate care that nurses provide. The findings of this scoping review will be relevant for nurse educators, administrators, health care organizations, nursing regulatory bodies, and nursing professional groups preparing nurses for practice in an era of AI. Furthermore, it will provide valuable information on roles of nurses in the co-design of AI-driven digital health technologies. Results of this review will be disseminated in a briefing paper, which will be used to inform a symposium organized by Associated Medical Services Healthcare in partnership with the RNAO.

## Appendix

Multimedia Appendix 1 Search terms.

## Abbreviations

AI: artificial intelligence.
CINAHL: Cumulative Index of Nursing and Allied Health Literature.
PRISMA-ScR: Preferred Reporting Items for Systematic Reviews and Meta-Analyses Extension for Scoping Reviews.
RNAO: Registered Nurses’ Association of Ontario.
